# Efficient Online Recruitment of Patients With Depressive Symptoms Using Social Media: Cross-Sectional Observational Study

**DOI:** 10.2196/65920

**Published:** 2025-06-03

**Authors:** Carolin Haas, Lisa Klein, Marlene Heckl, Marija Kesić, Ann-Katrin Rueß, Jochen Gensichen, Karoline Lukaschek, Tobias Kruse, POKAL-Group

**Affiliations:** 1 Institute of General Practice and Family Medicine University Hospital, LMU Munich Munich Germany; 2 "POKAL (Predictors and Outcomes in Primary Care Depression Care) Graduate Program” (DFG-GrK 2621) Munich Germany; 3 Trials24 GmbH, SubjectWell Munich Germany; 4 Independent researcher (Psychiatry) Munich Germany; 5 See Acknowledgments

**Keywords:** depression, suicidality, online patient recruitment, social media recruitment, digital patient recruitment, artificial intelligence

## Abstract

**Background:**

Over 80% of trials worldwide fail to complete patient recruitment within the initially planned time frame. Over the past decade, the use of social media for recruitment in medical research has become increasingly popular. While Google and Facebook are well established, newer social media channels such as Instagram and TikTok garner less research attention as recruitment tools. Although some studies have investigated the advantages and disadvantages of using social media for recruitment, a considerable gap still exists in understanding the precise mechanisms and factors that make different social media platforms most effective and cost-efficient for patient recruitment in mental health studies.

**Objective:**

This study evaluates the effectiveness of recruitment strategies implemented during the investigative phase of a validation study for a new suicidality assessment questionnaire optimized for primary care.

**Methods:**

We describe how online recruitment contributed to the enrollment of patients with depressive symptoms for the validation of a suicidality questionnaire (Suicide Prevention in Primary Care), which required over 500 participants. To this end, we analyzed differences in sample demographics between traditionally recruited and online participants, compared advertising metrics and conversion rates, and conducted a cost-benefit analysis.

**Results:**

We found online recruitment to be a fast and efficient method of securing the required number of participants with depressive symptoms for the study and increasing patient diversity. Considering the distribution of gender, age, and Patient Health Questionnaire-9 scores, participants recruited offline and online were equally eligible for the study. Online recruitment demonstrated high advertising efficiency. For example, the study population responded well to video advertisements on social media; these performed 50% to 70% more cost-efficiently than the best image advertisements. Moreover, a long website copy proved slightly better than a short version. Pixel tracking for improved advertisement targeting reduced advertising costs per suitable participant by 83.3%, making the advertisements 6 times more cost-efficient.

**Conclusions:**

Social media recruitment increased the diversity of patients in the studies and proved suitable for vulnerable and hard-to-reach populations. The total cost per patient recruited online was comparable to that achieved using offline methods, but overall recruitment progressed faster. In this study, implementing video advertisements and pixel tracking resulted in significant cost savings.

## Introduction

### Background

In primary care, approximately 10% of patients experience suicidal ideation [1]. Studies indicate that these individuals often seek contact with their general practitioners (GPs) during suicidal crises [2-5] but rarely report their suicidal thoughts on their own initiative [6,7]. Moreover, GPs often feel overwhelmed when interacting with patients with suicidal tendencies [8,9]. Thus, a new short questionnaire for suicidal assessment, Suicide Prevention in Primary Care (SuPr-X), was designed and validated in various treatment settings, optimized for primary outpatient care, and aimed at guiding health care professionals when interacting with patients with suicidal tendencies. Detailed information about the development [10,11] and validation [12] of the questionnaire can be found elsewhere.

However, recruiting the required number of patients for the SuPr-X validation study proved to be a significant obstacle. In medical and psychological research, generating valid study results by collecting data from large representative samples is essential [13]. For many research teams, recruiting the number of participants needed on time and within budget limitations becomes a major challenge [14]. Over 80% of trials worldwide fail to complete patient recruitment within the initially planned time frame, with insufficient case numbers being the primary reason for discontinuing 55% of all studies [15].

Studies in outpatient care rely on physicians’ practices to recruit patients. Conventionally, the first step involves finding collaborative practices that are interested in the research topic [16]. Participating physicians then screen suitable patients and recruit them for the study using leaflets, posters, or directly approaches to potentially eligible patients. Here, two worlds often collide: the interest and willingness of medical practices to actively recruit and the constant pressure to provide care coupled with a lack of time [16]. In studies specifically within the primary care setting in the United Kingdom, less than a third of the studies managed to meet the recruitment time frame: recruitment challenges mainly stem from the GP having to shoulder the responsibility of handling patient consent and inclusion [17]. To alleviate this bottleneck, researchers need to identify different solutions.

In the last 10 years, the use and popularity of social media recruitment in medical research has gained popularity [18]. Targeted advertisements on commonly used search engines such as Google are also often used to extend visibility and cover a more extensive outreach to potential participants to complement traditional recruitment methods, such as email or phone lists, prints, and leaflets [19-21]. Despite the growing use of social media for patient recruitment in mental health studies, the effectiveness and cost-efficiency of Instagram and TikTok remain underexplored. While Google and Facebook are well-established methods, studies on newer platforms such as Instagram are limited and primarily focus on younger audiences [22,23]. Social media provides the opportunity to reach a broad and diverse target audience and is helpful for highly focused recruitment approaches in a hard-to-reach population [18]. The time and costs involved are generally significantly lower than those associated with traditional recruitment methods [18,24-26]. However, potential limitations in representativeness must be considered [27]. When explicitly focusing on mental health studies (n=176), social media recruitment is indeed a cost-effective approach [28]: It was primarily used in studies including substance abuse (43.8%), mood disorders (15.3%), general mental health (11.9%), trauma and stress-related disorders (8.9%), and suicidal ideation and self-injurious behaviors (4.5%). In around two-thirds of the studies, social media performance was just as good or superior to traditional strategies such as mailing lists or referrals [28]. Moreover, participant characteristics from patients recruited online only differ minimally compared to those recruited from general practices, as a study on urinary incontinence found [29]. A web-based mental health program study showed that social media had the highest reach, lowest cost per participant, and similar adherence rates compared to general practice and pharmacies [30]. Although some studies have explored the advantages and disadvantages [18,24-28], there is still a notable gap in understanding the precise mechanisms and factors by which various social media platforms can be most effectively and cost-efficiently used for patient recruitment in mental health studies.

At the beginning of the SuPr-X study in 2022, we could not motivate GPs to cooperate due to the increasing spread of the highly contagious COVID-19 Omicron variant [31]. Conducting research on other topics became increasingly challenging [32]. It was not until spring 2023 that the situation improved, and study recruitment accelerated, resulting in some patients being included in practices. However, by this time, any buffer for recruitment delays had been exhausted, making the recruitment target of 500 seem no longer achievable by the previously used conventional method. Starting in September 2023, we used an online recruitment strategy to support the project.

### Objective

This study aims to evaluate the effectiveness of recruitment strategies in validating a suicidality assessment tool for primary care settings.

## Methods

### Study Design

This study used a cross-sectional observational design. For the SuPr-X validation study, we recruited adult patients with depressive symptoms from outpatient settings (GPs and psychotherapeutic or psychiatric practices) and from partial or full inpatient settings and online (subsequently enrolled on-site). The inclusion criteria required a Patient Health Questionnaire-9 (PHQ-9) score >5 and the ability to complete a 30-minute German-language questionnaire. Suicidal thoughts were not mandatory. People were excluded from the study if they had cognitive impairment, had significant positive psychotic symptoms (eg, hallucinations or disorganized thinking), or were minors.

### Measures

#### PHQ-9 Score

The PHQ-9 assesses depression severity over the past 2 weeks using 9 items (on a scale from 0=not at all to 3=nearly every day). The instrument has been validated in German, including evaluation for primary care use, and exhibits strong psychometric properties [33,34]. The PHQ-8 is a short form that does not contain the item related to death wishes or suicidality from the PHQ-9 [35]. PHQ-9 severity can be classified as minimal (>0), mild (≥5), moderate (≥10), moderately severe (≥15), and severe (≥20) [36].

#### Beck Suicide Ideation Scale

The Beck Suicide Ideation Scale (BSS) is based on a self-assessment of the past week using 19 items and a 3-point scale. Part 1 (items 1-5) serves as a screening tool. All further items are completed if the patient expresses active suicidal ideation (item 4=desire to kill oneself) or passive suicidal ideation (eg, if they prefer not to make apparent attempts to save themselves in the event of a life-threatening situation; item 5) [37].

#### SuPr-X Scale

The preliminary version includes 17 items. Four items (a-d) inquire about lifetime risk factors, such as family history of suicides. The response options are dichotomous (yes or no), and if a question is answered affirmatively, it prompts a brief open follow-up. Four items (1-4) explore protective or moderating aspects, such as confidence. Moreover, 7 items (5-11) assess current suicidal tendencies, such as ideation, plans, or suicidal behaviors. The response options for these are provided on a 4-point scale ranging from 0 to 3, with 0 indicating “strongly disagree,” 1 indicating “somewhat disagree,” 2 indicating “somewhat agree,” and 3 indicating “strongly agree.” The final item (item 12) asks a dichotomous question about whether there has been a suicide attempt in the last 2 weeks (yes or no). If the answer is no, it is followed up with multiple-choice options detailing reasons that prevented a suicide attempt (item 12.1). This structure situates the risk scale between protective items, facilitating a supportive start and conclusion to the interview by offering a perspective [12].

### Patient Recruitment Settings and Strategies

#### Overview

We used traditional recruitment strategies (direct approach by physician or therapist, leaflets with subsequent enrollment in collaborating practices and clinics) from July 2022 to February 2024, as well as social media and online advertisement (via Facebook [Meta Platforms, Inc]; Instagram [Meta Platforms, Inc]; TikTok [ByteDance Ltd]; and Google Ads [Google LLC] with subsequent enrollment at the Institute of General Practice and Family Medicine in Munich, Germany), starting from September 2023 to February 2024. Traditional recruitment took place in 20 general practices in Germany and Austria and 12 psychotherapeutic practices. In addition, recruitment occurred in 4 inpatient psychiatric wards and 2 daycare clinics. We contacted practitioners at events and conferences via telephone lists or email to raise awareness about the ongoing study. If there was interest, we delivered the study materials for implementation. The practitioners recruited suitable patients with depressive symptoms themselves. For this purpose, we provided them with leaflets and posters for waiting rooms. Patients who became aware of the study through an online campaign and met the inclusion criteria (online prescreening questionnaire PHQ-8) had the opportunity to schedule an appointment at the Institute of General Practice and Family Medicine either through a publicly shared calendar or with the help of a telephone patient service. Study psychologists or medical physicians performed an informative consultation on the study appointment and repeated the inclusion criteria check. Afterward, participants received the questionnaire from a study employee to complete independently on-site. The results were subsequently reviewed and discussed with the patients, particularly concerning questions on current suicidal tendencies. Further details about the recruitment process are given in Multimedia Appendix 1.

#### Sample

The sample included in the study through traditional recruitment methods (n=282, 54% of the total study population) consisted of patients recruited by GPs (n=105, 37.2%), patients recruited by psychotherapists (n=50, 17.7%), patients from a psychosomatic day clinic (n=74, 26.2%) and patients from an inpatient psychiatric setting (n=53, 18.8%).

Patients recruited online and subsequently invited to the Institute of General Practice and Family Medicine to participate in the study (recruited online but enrolled on-site) were questioned about their treatment and whom they consider their main physician or therapist. Of the 239 online recruited patients (46% of the total study population, N=521), we divided them into 4 groups: 230 (96.2%) patients reported having a GP, with 106 (46.1%) indicating that their GP was their main practitioner and was most familiar with their medical history. In total, 131 (54.5%) of the 239 online recruited patients were connected to psychiatric services, of whom 51 (38.9%) regarded the psychiatrist as their main practitioner. Moreover, 118 (49.4%) of the 239 online recruited patients received psychotherapy, with 66 (55.9%) viewing the psychotherapist as their main practitioner. Furthermore, 51 (21.3%) of the 239 online recruited patients regularly interacted with other physicians and therapists (eg, neurologists and pain specialists), of whom 17 (33%) considered these professionals as their primary medical contact.

### Technical Setup of Online Advertising, Study Website, and Online Prescreening Questionnaire

We used Google Ads Manager, AdEspresso (Facebook and Instagram advertisements management), and TikTok Ad Manager to set up the online campaigns targeted at Munich and nearby areas (a radius of 40 km). We implemented tracking pixels as described by the respective advertisement platforms. The advertisements forwarded interested users to the study website [38]. As per the General Data Protection Regulation, we implemented a cookie consent management platform (Borlabs Cookie). Only if users explicitly consented to advertising cookies did we track and include them for website statistics analysis. On the study website, the online prescreening questionnaire was embedded via HTML iframe (survey provider: Lamapoll [Lamano GmbH & Co KG], all data stored on German servers). Website visitors were screened with the PHQ-8 to ensure study participants showed at least mild depressive symptoms (cutoff >5). Three distinct questionnaire exit pages measured the outcomes of the prescreening questionnaire. They were used for conversion tracking of participants who (1) were eligible and left their contact data (“eligible opt-in”), (2) were eligible but did not leave their contact data (“eligible no opt-in”), and (3) were not eligible (“not eligible”).

We defined the desired conversion action as a user passing the prescreening questionnaire and providing their contact details (tracking done using the web page “eligible opt-in”); hence, a conversion represented an eligible patient lead. Throughout answering the online survey, participants remained entirely anonymous. Only the outcome page was then tracked again. Thus, end-to-end tracking from click to enrollment was not possible without the user’s consent; their data were transferred to Trials24’s system only when the participants passed the prescreening questionnaire, explicitly consented to the data protection declaration, and entered their contact details. We calculated the number of eligible patient leads passing the prescreening questionnaire to fill in their contact details by the respective tracking pixel’s measurement (“tracked” leads) or counting the entries in our database (“DB” leads). For some advertisement experiments, for example, pixel versus no pixel, additional versions of the website and the prescreening questionnaire were created so traffic from online advertisements could be directed to a specific version of the web page and prescreening questionnaire, allowing distinct measurements even without conversion tracking.

The online prescreening questionnaire did not include a special captcha question for participants to prove they were human, but the contact center manually checked every contact in the database.

### Creative Material Used for Online Advertising

#### Images

The following images were used (details are given in Multimedia Appendix 2): (1) a sad woman lying on the bed looking into the camera—gray theme with an orange textbox (“together against depression”); (2) a sad man sitting on the couch looking downwards—sepia theme with an orange textbox (“together against depression”); (3) a senior male physician looking into the camera with folded arms—white and blue theme with an orange textbox (“together against depression”); (4) couple 1, a couple with a woman in front looking concerned and sad—gray theme with an orange textbox (“together against depression”); (5) couple 2, a couple with a man in front looking concerned and sad—gray theme with an orange textbox (“together against depression”);

#### Video

We used 2 versions of the same video: (1) a female influencer dressed in everyday clothes explaining the study and (2) a female influencer dressed as a medical physician explaining the study. The person who recorded the video is a medical influencer who talks about medical topics, for example, on TikTok. The influencer has neither published the video on her personal channel nor mentioned her profession to ensure that only the visual impression differs.

### Statistical Analysis

The association between the recruitment method and gender distribution within the different samples was analyzed using the φ coefficient for nominally scaled items. With a 2-tailed *t* test, we determined if there were group differences regarding the recruitment method (traditional versus online) and age. Nonparametric tests are widely recommended when the assumptions of parametric tests are violated, such as interval scaling and normal distribution. Therefore, they are particularly suitable for ordinally scaled data, which are often found in psychological or medical questionnaires [39]. Data were not normally distributed for each online recruitment channel (Shapiro-Wilk test, *P*<.05); therefore, we chose a nonparametric test: the Kruskal-Wallis test was performed to examine whether age differences exist depending on the online recruitment channel. A nonparametric Mann-Whitney *U* test for ordinally scaled items was conducted to assess possible group or subgroup differences regarding depression symptoms measured by PHQ-9. We also used the Mann-Whitney *U* test to determine possible differences in reported suicidality between recruitment methods measured by the SuPr-X Risk scale (sum score of items 5-11) and BSS screening (sum score of items 1-5). For pairwise comparisons and post hoc tests, Bonferroni-adjusted *P* values were reported to account for multiple testing. For nonparametric tests, the rank-based effect size *r* was used, calculated by dividing the test statistic’s *z* statistic by the square root of the total number of observations (N), with divisions into small (0.1-0.3), medium (0.3-0.5), and large (>0.5) effects [40]. To compare the effectiveness of recruitment methods, a linear regression analysis was performed to estimate the monthly participant enrollment rates (slopes) for the traditional and online recruitment methods. We quantified the magnitude of differences between slopes using Cohen *d*, calculated as the difference between group means or slopes divided by the pooled SD, with Cohen *d*=0.2 (small), Cohen *d*=0.5 (medium), and Cohen *d*=0.8 (large) [40]. Any *P* value <.05 indicated statistical significance. We used SPSS (version 29; IBM Corp) for all calculations.

### Ethics Approval

The study and all recruitment activities received ethics approval from the medical ethics committee of Ludwig-Maximilians-Universität München (Munich) on May 9, 2022 (project number 22-0028) and were performed in accordance with applicable guidelines and regulations. The amendment for additional online recruitment was approved on August 8, 2023. All participants signed the informed consent on-site after an informed prestudy briefing with the respective clinician from the cooperating practice or, in the case of online recruitment, on-site at the Institute of General Medicine with the responsible study physicians or psychologists. All data were securely stored, and access to data storage was restricted to authorized personnel only. All participants received a €25 shopping voucher as compensation. The conversion rate (CR) at the time of the study was €1=US $1.074.

## Results

### Sample Characteristics

From July 2022 to February 2024, a total of 521 patients with depressive symptoms (inclusion criteria: PHQ-9 >5 “mild depression”) were included in the study: 67.8% (n=353) women, 31.7% (n=165) men and 0.38% (n=2) nonbinary participants (n=1 missing, 0.19%). The mean age was 40.89 (SD 14.32) years, and the average PHQ-9 score was 14.8 (SD 4.97), indicating moderate severity [36].

After presenting the overall sample characteristics, we examined how traditional and online recruitment strategies influenced differences within the sample.

### Differences in Sample Characteristics Based on Traditional Recruitment Strategies Versus Online

The online recruitment strategy focused on outpatient demographics. For reasonable comparative analysis of sample characteristics using traditional versus online recruitment methods, the inpatient psychiatric cohort was excluded to avoid biases. However, online recruitment included an *outpatient* psychiatric group, which did not have a corresponding comparative group within the traditionally recruited cohort (only the previously mentioned excluded *inpatient* psychiatric group). As a result, a feasible comparison of sample characteristics among the subgroups “traditional” versus “online” could only be conducted within the GP and psychotherapist samples, as detailed in Table 1. Moreover, 74 (14.2% of the 521 patients) from the psychosomatic day clinic were considered for the *total* result but are not separately reported in the table.

Table 1 shows gender, age, depressive symptoms mean scores (PHQ-9), and suicidality mean scores (SuPr-X risk: 5-11; BSS screening: 1-5) of the traditional sample in comparison to the online recruitment sample. Subgroup comparison of participants are attributed to GPs and psychotherapists.

In summary, we recruited participants through traditional methods and online (Table 1), with neither significant difference in gender distribution across recruitment methods (φ=0.051; *P*=.54) nor age differences (t_465_=–0.1886; *P*=.06). Depression scores also did not vary significantly between groups (*U*=26,314.500; *Z*=–.479; *P*=.63). We observed an increased suicide risk in the online recruitment group (SuPr-X: *U*=22,969.500; *Z*=−3.025; *P*=.002 and BSS screening: *U*=23,611.500; *Z*=–2.323; *P*=.02) with small effect sizes (SuPr-X: *r*=0.140; BSS screening: *r*=0.108), as reflected in significantly higher scores on SuPr-X by 1.04 (95% CI 0.41-1.68) points and on BSS screening by 0.53 (95% CI 0.13-0.94) points. However, no significant difference was found in subgroup comparisons (GP and psychotherapist), indicating that the higher rates of suicide risk in the online recruitment sample were attributable to the psychiatric outpatient sample.

Building on the comparison of traditional and online strategies, we further explored differences in sample characteristics across specific online recruitment channels.

**Table 1:** Sample comparison of traditional recruitment strategies versus online.

		Traditional recruitment				Online recruitment			
		Total	GP^a^	Psychotherapist	Total		GP	Psychotherapist	Psychiatrist
**Gender**									
	Total, n	229	105	50	239		106	65	51
	Men, n (%)	69 (30.1)	29 (27.6)	14 (28)	77 (32.2)		38 (35.8)	19 (29)	14 (28)
	Women, n (%)	160 (69.9)	76 (72.4)	36 (72)	161 (67.4)		68 (64.2)	45 (69)	37 (73)
	Nonbinary, n (%)	0 (0)	0 (0)	0 (0)	1 (0.4)		0 (0)	1 (2)	0 (0)
**Age (y)**									
	Total, n	228	105	50	239		106	65	51
	Mean (SD; range)	40.25 (14.74; 18-83)	43.67 (15.79; 18-83)	38.08 (14.15; 20-72)	42.75 (13.99; 18-77)		43.82 (14.35; 18-75)	38.08 (12.99; 18-73)	45.41 (14.13; 18-77)
**PHQ-9^b^**									
	Total, n	226	104	50	239		106	65	51
	Mean (SD; range)	14.47 (4.58; 5-25)	14.93 (4.416; 5-25)	13.40 (5.474; 5-25)	14.35 (5.044; 5-27)		13.87 (5.05; 5-26)	13.92 (5.25; 0-17)	15.57 (4.52; 6-26)
**SuPr-X^c^ risk**									
	Total, n	229	105	50	236		104	65	50
	Mean (SD; range)	1.73 (2.94; 0-15)	2.14 (3.35; 0-13)	1.56 (2.44; 0-9)	2.80^d^ (3.99; 0-17)		2.51 (3.92; 0-17)	2.51 (3.80; 0-17)	3.94 (4.34; 0-16)
**BSS^e^ screening**									
	Total, n	226	102	50	237		105	65	50
	Mean (SD; range)	1.48 (1.97; 0-8)	1.72 (2.28; 0-8)	1.52 (1.79; 0-6)	2.01^d^ (2.44; 0-10)		1.75 (2.36; 0-10)	2.12 (2.49; 0-10)	2.42 (2.54; 0-9)

^a^GP: general practitioner.

^b^PHQ-9: Patient Health Questionnaire-9.

^c^SuPr-X: Suicide Prevention in Primary Care.

^d^Significant group differences (Suicide Prevention in Primary Care: *P*=.002; Beck Suicide Ideation Scale: *P*=.02).

^e^BSS: Beck Suicide Ideation Scale.

### Differences in Sample Characteristics Based on Online Recruitment Channels and Search Engines

Online recruitment channels were classified as (1) Facebook, (2) Instagram, (3) TikTok, (4) Google, (5) internet, and (6) others. The category “internet” included all responses from participants that could not be attributed to a specific online platform or search engine. “Others” included responses from participants indicating they learned about the study through people in their networks who had seen the study advertised online.

There was no statistically significant association between online recruitment platforms and gender distribution (φ=0.188; *P*=.21). The group recruited via TikTok (median 23, SD 9.238) was significantly younger than those recruited through Facebook (median 47, SD 11.479; Z=5.410; *P*<.001); Google (median 46, SD 15.005; *Z*=–4.670; *P*<.001); and other internet sources (median 52, SD 14.614; *Z*=–5.308; *P*<.001), with medium (Facebook *r*=0.489) to strong (Google *r*=-.704; internet *r*=0.766) effect sizes.

**Figure 1:**
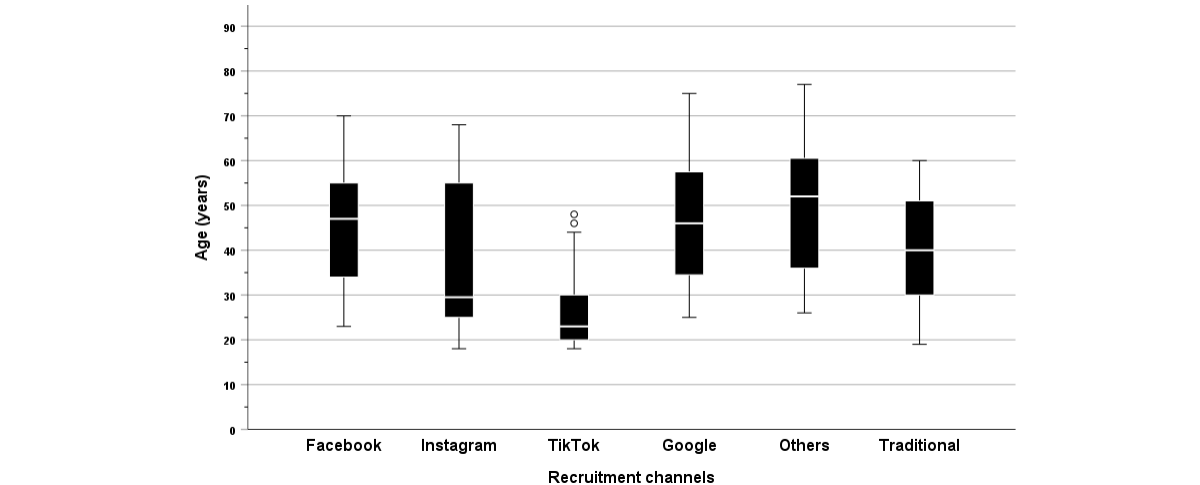
Age distribution across recruitment channels.

The mean age differences were –18.26 (SD 11.479, 95% CI –22.80 to –13.72) years for Facebook, –20.40 (SD 15.005, 95% CI –27.70 to –13.10) years for Google, and –21.94 (SD 14.614, 95% CI –28.72 to –15.16) years for other internet sources. Age did not differ significantly from Instagram (median 29.5, SD 16.796; *Z*=2.038; *P*=.62). These age differences suggested that TikTok may attract a younger demographic for online study recruitment (Figure 1). The severity of depression symptoms did not depend on the online recruitment platform (Kruskal-Wallis H_5_=8.773, *P*=.12).

Box plots illustrate the median age and the lower and upper quartiles, minimum, and maximum values across all online recruitment channels compared to the traditional recruitment sample.

### Enrollment Timeline

**Figure 2:**
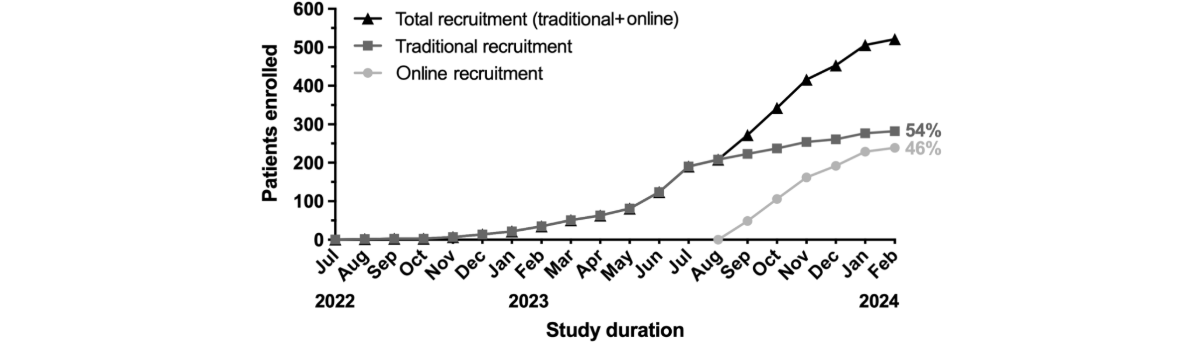
Number of participants included in the study. The black line displays the total recruitment timeline. Traditional recruitment (dark gray line, squares) was active from July 2022 to February 2024. Online recruitment was activated in September 2023 and is shown as the light gray line (with circles).

The comparison of recruitment methods revealed a highly significant difference in participant enrollment rates (Figure 2). Linear regression analysis estimated a slope of 17.95 (95% CI 15.44-20.46) for the traditional recruitment method and 41.54 (95% CI 32.74-50.33) for the online campaign. The difference between the slopes was 23.59 (SE 3.62), which was highly significant (*P*<.001; *Z*=6.51). The effect size, expressed as Cohen *d*, was 0.22, indicating a small to moderate practical effect. In addition, the correlation coefficient *r*=0.79 reflects a strong relationship between the recruitment methods and their differences in performance.

Building on the timeline analysis, we illustrate the process of converting clicks into enrolled participants, noting that comparable data for the traditional setting were not collected.

### CRs in Online Recruitment

**Figure 3:**
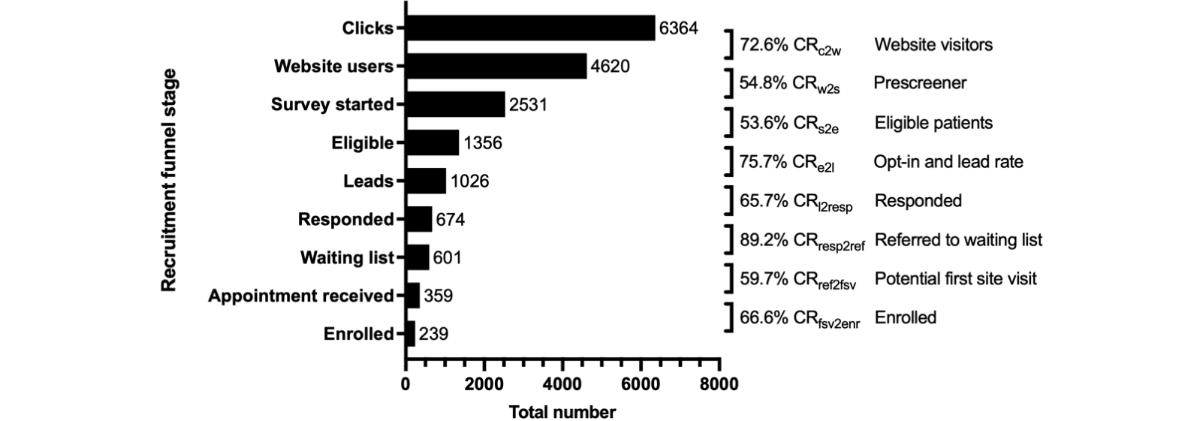
The patient journey to enrollment in the online recruitment funnel for Suicide Prevention in Primary Care. CR: conversion rate; CRc2w: CR from click to website user; CRe2l: CR from eligible to lead; CRfsv2enr: CR from first site visit to enrolled; CRl2resp: CR from lead to responded; CRref2fsv: CR from referral to first site visit; CRresp2ref: CR from response to referral; CRs2e: CR from survey to eligible; CRw2s: CR from website to survey.

Figure 3 describes the typical patient journey during online patient recruitment, the “patient journey to enrollment.” The advertisements were displayed 890,145 times (impressions), and 6364 (0.7%) people showed interest by clicking on the study advertisement. In addition, 72.6% (4620/6364; CR from click to website) of website users landed on the study website, and 54.8% (2531/4260; CR from website to survey) individuals started the online prescreening survey. In addition, a 53.6% (1356/2531) CR from survey to eligible respondents was met for the study-specific inclusion criteria, making them potentially eligible and reaching the end of the questionnaire. Of those 1356 individuals, 1026 individuals (75.7% opt-in rate; CR from eligible to lead=opt-in rate) left their contact data, becoming an eligible “lead.” When the patient companion service*—*a dedicated contact center for interested patients*—*reached out to them, 65.7% (674/1026) CR from lead to people responded, and an 89.2% (601/674) (CR from response to referral=potential referral rate) were placed on the study waiting list because there were only limited slots available for participation.

Overall, 59.7% (359/601) of individuals (CR from referral to first site visit=potential first site visit rate) received an appointment slot. There were reminders 2 days and 1 day before the appointment so participants could cancel their appointment in time if they could not attend (appointments were rescheduled, and open slots were given to the next person on the waiting list). Only 5.3% (19/359) of individuals were listed as “no-shows.” Of those who attended their appointment, 66.7% (239/359; CR from first site visit to enrolled) of individuals were subsequently enrolled.

The patient journey to enrollment in the online recruitment funnel starts with the first click on the study advertisement and then continues to the website visit, before qualification via the online prescreening questionnaire, making an appointment with the patient companion service, and finally ends with participation in the study by signing the informed consent form and being enrolled. The following conditions apply: clicks=registered click on a study advertisement; website users=people on the study website; survey started=people who have started the online prescreening questionnaire; eligible=people who showed potential eligibility online; leads=people who have provided their contact details after being potentially eligible; responded=people who responded when the patient companion service reached out to them; waiting list=all people who wanted to receive an appointment for the study; appointment received=people who received an appointment slot; enrolled=people who completed the study according to the study protocol.

Beyond CRs, we also presented detailed online advertising efficiency metrics to comprehensively assess cost-effectiveness.

### Online Advertising Efficiency Metrics

#### Overview

We chose Google, Meta (Facebook and Instagram), and TikTok as advertising platforms. We launched 13 distinct advertisement campaigns (8 in Facebook, 2 in Instagram, 2 in TikTok, and 1 in Google) to perform experiments regarding different advertisement settings or creative material. The advertisement campaigns were set up to create maximum leads (details described in the Methods section). Table 2 shows the key metrics and costs per channel for study recruitment.

When comparing traditional and online recruitment, the overall recruitment cost per enrolled patient (CPE) were €30 and €29.21, respectively. However, online patients still needed to be screened on-site. The CPEs between different online platforms varied between €23.08 and €52.49.

Three metrics can be used to compare advertisement efficiencies of different channels: cost per lead (CPL) tracked as measured by the advertisement platform (but sensitive to cookie consent, browser tracking blockers, user settings, etc), CPL calculated as the cookie consent corrected CPL tracked, and CPL database as the cost per registered person based on their reported source. In theory, CPL calculated and CPL database should be the same, as they reported the same metric but were based on different data sources. For Google and Facebook, this seemed accurate, as the CPLs only differed by approximately 10%. Instagram and TikTok CPLs fluctuated by 60%, indicating that numbers from conversion measurement and participant-reported advertisement channels did not match.

**Table 2:** Overview of advertising metrics for study recruitment.

	Cost (€^a^)	Leads tracked^b^, n	Leads calculated^c^, n	Leads database^d^, n	Enrolled^e^, n	Cost per lead tracked	Cost per lead calculated (€)	Cost per lead database (€)	Cost per enrollment (€)
Google^f^	750.20	38	53	59	16	19.74	14.04	12.72	46.89
Facebook	4346.75	351	529	470	116	12.38	8.22	9.25	37.47
Instagram^g^	600	72	112	187	26	8.33	5.38	3.21	23.08
TikTok	1049.81	103	162	97	20	10.19	6.46	10.82	52.49
Other^h^	—^i^	—	—	182	53	—	—	—	—
Total online	6746.76	564	856	995	231	11.96	7.88	6.78	29.21
Traditional: practitioner’s compensation	8460	—	—	—	282	—	—	—	30.00

^a^The conversion rate at the time of the study was €1=US $1.074.

^b^Leads (tracked)=conversion defined as eligible patient lead measured by advertising platforms

^c^Leads (calculated)=calculated number of participants that left their contact details when conversions are corrected by the overall cookie consent rate,

^d^Leads (database)=participants that left their contact details in the database and reported to have clicked on a particular advertisement

^e^Enrolled=reported advertisement source by enrolled participants of the study.

^f^First 3 days of the Google advertising campaign were excluded from the metrics (incorrect settings).

^g^Instagram advertisements were run only with the best-performing creative material.

^h^Other=participants in the database that could not be attributed to Google, Facebook, Instagram, or TikTok.

^i^Not available.

#### Additional Expenses

For online recruitment, the average time per participant was approximately 24 minutes, including 9 minutes for telephone services and prescreening interview (€3 in personnel expenses) and 15 minutes for on-site inclusion and debriefing by the psychologist and doctoral researcher (€6.66 in personnel expenses). This totals 24 minutes and €9.66 per participant, plus advertising costs for the social media campaigns.

In comparison, traditional recruitment by practitioners involved a flat compensation of €30 per participant. On the basis of typical German fee schedules (German Uniform Assessment Standard EBM code 03230 [41], €14.74 per 10 minutes, adjusted for fee increase between quarter 4 of 2022 and quarter 1 of 2024), a minimum time investment of 30 minutes per patient is estimated, resulting in a total cost of €44.22 per patient, including €14.22 in additional opportunity costs.

In summary, the estimated time and personnel costs per participant were 24 minutes and €9.66 for online recruitment, compared to 30 minutes and €14.22 for traditional recruitment. It should be noted that these are rough estimates, as the actual time spent in routine practice during traditional recruitment was not documented.

In addition to overall efficiency, we examined gender-specific differences within the target population to identify potential disparities in recruitment outcomes.

### Gender-Specific Differences in the Target Population

We used Facebook advertisements to compare the gender-specific differences in online campaigns. The advertisement spent on men and women was equal, but CPLs and CPE for women were only half as expensive. Metrics for women were as follows: CPL tracked=€9.17; CPL calculated=€6.20; CPL database=€6.32; and CPE=€26.48. The metrics for men were as follows: CPL tracked=€17.87; CPL calculated=€11.92; CPL database=€14.78; and CPE=€55.53. This matched the overall 2:1 ratio of women and men enrolled in the study (Table 1). The total online numbers for women were 725 leads and 160 enrolled, and the totals for men were 302 leads and 79 enrolled.

As we found gender differences in the recruitment of participants, we also wanted to find out whether there were key factors related to the creative material that influenced recruitment.

### Impact Factors on Online Patient Recruitment Efficiency

#### Use of Different Images

Most advertisements used a central image. First, we tested 5 different images on Facebook for their general performance. Initial results showed that the photos of the couples performed the least and thus were excluded from subsequent testing. The remaining 3 images were tested in an A/B split test, meaning they ran simultaneously, in the same audience, with the same advertisement budget. CPL tracked results for the male audience were €19.84 (sad woman), €24.79 (sad man), and €28.43 (senior male physician), while the female audience resulted in a CPL tracked of €7.76 (sad woman), €25 (sad man), and €13.44 (senior male physician). The image of the sad woman performed best in both audiences and achieved 20% to 30.2% and 42.3% to 69% lower costs in the male and female audiences, respectively.

#### Image Versus Videos

Furthermore, 3 images (sad man, sad woman, and senior male physician) and 2 videos (physician and layperson) were A/B tested on Facebook and Instagram (Table 3). On Facebook, CPL tracked was €16.21 (images) and €9.52 (videos), showing that image-based advertisements were 70.2% more expensive than video-based advertisements. When looking only at the best-performing image or video asset, the image of the sad woman performed best with a CPL tracked of €11.11, and the layperson video performed best with a CPL tracked of €8.

**Table 3:** Image versus video advertisements. We compared a standard image-based advertisement with a more modern video-based advertisement (N=1011).

Social media site			Cost per eligible patient lead (€^a^)
**Facebook**			
	Image	16.21	
	Video	9.52	
**Instagram**			
	Image	10.53	
	Video	6.90	
**TikTok**			
	Image	—^b^	
	Video	9.26	

^a^The conversion rate at the time of the study was €1=US $1.074.

^b^Not applicable.

On Instagram, the image of the sad woman and the video asset resulted in a CPL tracked of €10.53 for the image and a CPL tracked of €6.90 for the best-performing video (overall video CPL tracked of €7.55). Thus, also on Instagram, the video assets outperformed the best image by 52.6%.

TikTok exclusively allows videos as creative material; therefore, we could not test any images. Here, the overall video performance showed a CPL tracked of €9.26.

We saw that the 2 video variants performed differently on each social media channel, so we wanted to highlight this in more detail.

#### Appearance of the Influencer in the Video: Medical Physician Versus Layperson

On Facebook, Instagram, and TikTok, both videos—medical physician versus layperson—were tested against each other (Table 4). On Facebook, the video with the physician’s outfit was 47.1% more expensive than the layperson’s (CPL tracked physician €11.76 and layperson €8). Interestingly, the same video resulted in 17.2% and 15.9% lower CPLs on Instagram and TikTok, respectively (Instagram: CPL tracked physician €6.9 and layperson €8.33 and TikTok CPL tracked physician €8.52 and layperson €10.13).

**Table 4:** Appearance of the influencer in the video: medical physician versus layperson. We compared a video showing the influencer in a physician’s and a layperson’s outfits (N=176).

Social media site			Cost per eligible patient lead (€^a^)
**Facebook**			
	Physician	11.76	
	Layperson	8.00	
**Instagram**			
	Physician	6.90	
	Layperson	8.33	
**TikTok**			
	Physician	8.52	
	Layperson	10.13	

^a^The conversion rate at the time of the study was €1=US $1.074.

Besides the visuals, the structure and length of the landing page copy seem to play a critical role in converting potential participants after the initial advertisement engagement.

#### Long Versus Short Landing Page Copy

We compared the performance of a long intro text (4 paragraphs, 199 words) against a short intro text (2 paragraphs, 133 words). Both versions included the same key messages but were phrased differently. In this experiment, the algorithm was not forced to use the budget equally on both versions, resulting in sending more traffic to the better-performing long copy. Overall results for the long copy were €506.7 advertisement spent, 72 conversions, and CPL tracked of €7.04, while short copy showed €243.4 advertisement spent, 33 conversions, and a CPL tracked of €7.37. Even though 90% of website traffic came from mobile devices, the long copy version achieved 4.5% lower CPLs.

In addition to the obvious influencing factors, such as texts and design, we also aimed to determine the effect that technical settings can have.

#### Using the Facebook Tracking Pixel to Improve Advertisement Targeting and Lower CPLs

Tracking pixels enable conversion tracking, feeding these data back to the advertisement platform’s algorithms and allowing them to create artificial intelligence–based anonymous user group profiles that are more likely to convert, thus optimizing advertisement targeting and lowering the CPL. We wanted to know exactly how much this tracking technology lowers lead costs. Two similar advertisements with a budget of €300 each were A/B split tested, with 1 advertisement not using the Facebook tracking pixel and the other one using it (Multimedia Appendix 3). The cost per click (CPC) on the advertisements was €0.53 (n=561 clicks) without and €1.45 (n=206 clicks) with the tracking pixel, showing the advertisement algorithms targeted specific users that are more expensive to reach. Interestingly, the CPL database showed the opposite: the advertisement without the tracking pixel produced n=3 leads, resulting in a CPL database of €99.81, while the advertisement with the tracking pixel produced 18 leads, resulting in a CPL database of €16.63 (Multimedia Appendix 3 right graph). The tracking pixel improved the advertising algorithm’s targeting, which reduced the CPL by 83.3%, making the advertising 6 times more cost-efficient.

## Discussion

### Principal Findings

This study aimed to evaluate the effectiveness of recruitment strategies while validating a suicidality assessment tool optimized for primary care. We found that online recruitment accounted for 46% (239/521) of participants, and they were recruited within a short time frame, demonstrating its potential for reaching vulnerable populations. The samples recruited online and using traditional methods were comparable regarding key demographic and clinical characteristics. Recruitment costs for online methods were similar to traditional approaches, while enrollment was significantly faster. In addition, impact factors such as advertisement formats, landing page design, and pixel tracking substantially influenced recruitment efficiency.

### Representativeness of Our Sample

In our analysis, age, gender, and the inclusion criteria for depressive symptoms did not differ among patients based on different recruitment methods (traditional vs online). For ethical reasons, online advertising for the study only mentioned depression and not suicidal ideation. Both groups showed an overrepresentation of women compared to men in a ratio of 2:1, which, however, corresponds to the general gender-specific distribution of depression [42].

Regarding the representativeness of demographic factors (age and gender) and the inclusion criterion (depressive symptoms), patients recruited online were comparable to traditional strategies. Analysis of online recruitment channels showed that TikTok, in particular, targeted younger audiences and could be used strategically to attract a younger sample.

An argument in favor of using social media recruitment in mental health studies is an observed correlation between time spent on social media (*r*=0.11), addictive use (*r*=0.29), and depressive symptoms [43], as well as anxiety, low sleep quality, and reduced self-esteem in adolescents. This correlation appeared to be stronger within the female sample [44]. These findings align with our study, where a significant proportion of participants were recruited through social media, including younger people and women, highlighting the platform’s potential to engage vulnerable populations effectively. Consistent with previous suicide prevention studies, Facebook was excellent in generating a broad and favorable reach to attract participants for a survey [45]. However, similar to the findings by Whitaker et al [27] and Tang et al [46], young women were overrepresented. This aligns with broader research indicating that female participants may be more responsive to social media recruitment campaigns, potentially due to higher engagement with mental health–related content [43,44]. A frequently cited limitation when using social media is the representativeness of the collected data. Because targeting can be adjusted to a certain extent in these campaigns, social media advertising can specifically address underrepresented groups to increase patient diversity. Yet, advertising costs would have become more expensive.

Online-recruited patients appeared especially suitable for the validation study, as they reported higher average levels of (self-reported) suicidality than the traditionally recruited cohort, thus providing valuable data for validating our new suicidality questionnaire. The online cohort also included 10 individuals of the “under-the-radar” population, defined in suicidology as those who are mentally distressed and suicidal but have minimal or sporadic contact with the health care system and hence are often missed by preventive measures [46]. We identified this group as patients who confirmed suicidal ideations or behavior in the questionnaire but also stated they were not receiving medical treatment and had not reached out for help due to mental stress. Clinical services often face challenges in reaching individuals most in need of mental health care. A systematic review [46] found that nonreceipt of services was linked to factors such as being male, younger or older age, rural residency, and minority ethnicity. Those without access were also less likely to have a psychiatric diagnosis, a history of suicidal behavior, or contact with health services but more likely to use violent suicide methods. These findings highlight the need for tailored, multifaceted strategies to overcome barriers and engage diverse at-risk groups effectively [46]. However, no clear associations could be derived from our data in this regard. Nevertheless, the campaign successfully reached out to them, and they were enrolled in the study. Suicidality was not mentioned in social media advertising, yet it appeared to have reached a demographic that traditional methods might have missed. Enrolling such underrepresented patients underlines the role of social media campaigns to increase patient diversity in research studies and clinical trials.

### Costs

Raising awareness is not an issue when recruiting patients online, as you can use paid advertising to display your messages to a broad audience. The critical part is to find participants who click on the advertisement, complete the survey, and provide their contact information. A meta-analysis by Brøgger-Mikkelsen et al [47] showed that online recruitment costs were generally lower than offline recruitment, but costs varied greatly: the median price per enrollment for online recruitment strategies was US $72 (range US $4-$251), and the median price per enrollment for offline recruitment strategies was US $199 (range US $19-$839). In our study, both recruitment strategies—online and offline—resulted in similar costs of approximately €30 per enrollment, positioning them in the lower range of reported online and offline recruitment costs. This highlights the cost-efficiency of both approaches in our specific context. Even if additional expenses for personnel costs are included (approximately €9 to €15), the overall recruitment costs are still in the lower to middle range. Furthermore, enrollment was completed before we could fully use the optimized advertisements. This means using only the most cost-effective creative material and the advertising platform’s artificial intelligence–based targeting algorithm without our restrictions regarding A/B split tests. If we had recruited for a longer period and only run the most optimized and efficient advertisements, we estimate that it would have been 40% to 60% more cost-effective. Furthermore, our appointment slots were limited. Actual online recruitment costs might have been half the reported or even less if every participant on the waiting list could have been included. Surprisingly, only 5.3% (19/359) of individuals did not show up, indicating that the target population was eager to participate in the study. Given that depression primarily occurs with symptoms such as a lack of energy and motivation, the observed low no-show rate was a pleasant surprise.

### Impact Factors

When specifically looking at clinical trial recruitment, little is known about which factors impact advertisement efficiencies. These advertisements require ethically correct phrasing and must align with country legislation regarding advertising in health care. In Germany, this is, for example, the Heilmittelwerbegesetz (translating to “Health Services and Products Advertising Act,” a law that prohibits advertising for prescription drugs). You can apply some strategies from online marketing; however, the overall toolbox is restricted.

One factor influencing advertisement efficiency was the social media platform used for advertising, as each platform has its predominant age group [48]. A rule of thumb with social media platforms is that teenagers and young adults mainly use the newest and trendiest platforms. At the same time, older people only switch to these platforms later or do not switch at all, resulting in social media platforms “aging up.” We also saw this effect with the TikTok population being significantly younger, followed by a slightly older group on Instagram, and Facebook having the oldest users.

When comparing the images, it was interesting to see that even in the male audience, the image of the woman performed best. It must be mentioned that the stock photos of the sad woman and man did not display the same posture and color scheme, so comparability was somewhat limited. Yet, the observed effect could be a result of increased self-stigmatization in male depression [49].

The use of negative imagery, such as depictions of sad individuals, in participant recruitment raises ethical and methodological concerns. Research suggests such portrayals may perpetuate stigma, deter help-seeking behaviors, and reinforce harmful stereotypes [50,51]. Bennett [52] highlights the limitations of visual clichés, noting they oversimplify mental health conditions and may influence who participates in studies.

When looking at image and video advertising for clinical trials, we did not find any reference that specifically showed insights into how the creative material was designed and which effects resulted from it. In our case, videos outperformed classical image advertisements, even though we used a relatively simple video setting of a person explaining the content. All our texts and scripts followed established conversion copywriting rules adapted to the advertising platforms and legal and ethical compliance guidelines. Therefore, the information flow was always the same, only presented in a different format. Because clinical trials are a medical topic, we were interested in how well a video featuring a medical physician performs compared to a layperson. A systematic review analyzing the impact of a physician’s attire on patient perceptions discovered that 60% of patients preferred a white coat, especially among older patients [53]. However, in our study, older patients seemed to prefer casual outfits, at least on social media. We assume that young adults may already be used to seeing medical influencers on social media, as this generation is also informing itself more and more about health topics online [54].

A well-researched fact is that the online attention span is extremely short, with the first 10 seconds being crucial as to whether a user continues reading or leaves the website [55]. We were interested in which effect might be stronger: condensing the information to a minimum word count so users could see all relevant information without needing to scroll much on their mobile phones or having a longer version with each paragraph spanning one topic. In the small sample we tested, the longer copy worked slightly better for finding potentially eligible participants for the study, indicating it was not the number of words but the text quality that made a difference.

Finally, we gained a technical but essential insight regarding advertising efficiencies when comparing advertisements with and without a tracking pixel. CPC is often used as an indicator of the advertisement’s cost-efficiency, even though it does not show whether the generated traffic is indeed quality traffic [56] that leads to conversions (which is the actual study leads). The CPC without a tracking pixel was lower, but the CPL—the more critical outcome—was 6 times as high. Ethics committees assessing study recruitment materials sometimes struggle to permit this kind of tracking, even though users in the European Union can opt out of being tracked via cookie consent options. Our data clearly show the need to allow this pixel-based tracking technology; otherwise, researchers must invest significantly more capital to find eligible study participants. This creates an additional financial burden, especially for small research organizations or universities with limited resources and delays the studies. Patients may have to wait longer for potential treatment to be approved, or studies may be prematurely terminated due to not reaching recruitment goals.

Having discussed the key factors influencing recruitment outcomes, we now acknowledge the limitations of our study to provide a balanced interpretation of the findings.

### Limitations

In the traditional recruitment setting, clinicians approached patients during routine practice but did not document the total number of patients approached. Only data for included patients were submitted, making it impossible to calculate precise CRs compared to online recruitment. In the online setting, small sample sizes often constrained the subanalyses of the considered impact factors, precluding generalized recommendations concerning image versus video materials, pixel tracking, and how much informational content should be provided. Variations in the images’ graphical design may also contribute to whether a picture gains user attention (Multimedia Appendix 2). Stereotypical imagery may have introduced selection bias by attracting participants who identified with these portrayals while deterring others, limiting sample diversity.

The metrics CPL tracked and CPL calculated are influenced by each user’s tracking blockers and technical settings, while CPL database relies on the accuracy of the individual’s statement. As a result, both technical measurements and human assessment may be prone to error. The low no-show rate may be attributed to our reminders and a common selection bias of motivated participants by the patient companion service. Furthermore, it remains unclear to what extent the results would change in other settings, for example, due to cultural influences on the perception of a person with or without a physician’s coat in an advertisement.

### Conclusions

Online recruitment demonstrated high efficiency, enrolling 46% (239/521) of the study patients quickly and providing a diverse sample representative of the study’s objectives. This approach proved particularly effective in reaching vulnerable, hard-to-access populations and enhancing patient diversity. The comparable costs and faster recruitment rate relative to offline methods highlight the practical advantages of online campaigns. While key insights from tested impact factors—such as the superior cost-efficiency of video advertisements, longer landing pages, and pixel tracking—offer promising strategies for optimizing recruitment efforts, these findings are preliminary and based on limited data. Further research with larger datasets is necessary to generalize these results and refine recommendations for future studies.

## Appendix

Multimedia Appendix 1 Supplemental flowchart of the recruitment process.

Multimedia Appendix 2 Creative material used for image and video advertisements.

Multimedia Appendix 3 Advertisements with and without using the Facebook tracking pixel.

## Abbreviations

BSS: Beck Suicide Ideation Scale
CPC: cost per click
CPE: cost per enrolled patient
CPL: cost per lead
CR: conversion rate
GP: general practitioner
PHQ: Patient Health Questionnaire
SuPr-X: Suicide Prevention in Primary Care

## Data Availability

The data supporting this study’s findings are available from the DFG-GrK 2621/POKAL studies but, due to licensing restrictions, are not publicly available. However, the data are available upon reasonable request. Data requests concerning the study population may be directed at Stiftung Allgemeinmedizin—The Primary Health Care Foundation [57]. Data requests concerning the results of online recruitment efficiency and the following may be directed to Trials24, a SubjectWell Company [58].
