# Supplementary material for: Efficient Online Recruitment of Patients With Depressive Symptoms Using Social Media: Cross-Sectional Observational Study
Source: JMIR Ment Health. 2025 Jun 3;12:e65920. doi: 10.2196/65920 (PMC12174873; doi:10.2196/65920)
Supplement: Multimedia Appendix 1 [file mental_v12i1e65920_app1.pdf]

Patient recruitment process

Online recruitment process

1. The patient is directed to the study website, where they can find information and participate in a survey to check eligibility for the study.
2. Screening includes questions to:
   - Age (18+),
   - Depression diagnosis
   - PHQ-8 score, and
   - Willingness to visit the Institute of General Medicine in Munich.
3. If eligible (18+, PHQ-8 > 5, willingness to visit the study center), the patient can leave their contact details.
4. The contact center will conduct a phone call with the patient to address questions and schedule an appointment.
5. A 15-minute slot with a psychologist/doctoral researcher is scheduled at the Institute for General Medicine to:
   - Review study information,
   - Conduct an informed consent discussion, and
   - Obtain written consent.
6. Upon consent, the patient independently completes the study questionnaires.
7. A joint review of the completed questionnaires is conducted, including a brief risk assessment by the psychologist.
8. Risk outcomes:
   - 8a: If minimal risk is assessed: a €25 voucher is provided, emergency contacts are shared, and the patient is discharged.
   - 8b: If moderate to severe risk is assessed: a €25 voucher is provided, and the patient is encouraged to visit the psychiatric outpatient clinic. The psychologist escorts the patient to the on-call doctor if necessary.

Traditional recruitment process

1. The clinician identifies suspected depressive symptoms in the patient.
2. The clinician informs the patient about the study and assesses their interest in participation.
3. Screening is conducted using the PHQ-9 questionnaire.
4. If eligible (PHQ-9 > 5 or suicide item > 0):
    - An informed consent discussion is conducted, and
    - Written consent is obtained.
5. The patient independently completes the study questionnaires.
6. A joint review of the completed questionnaires and a brief risk assessment is conducted by the clinician.
7. Risk outcomes:
    - 6a: If minimal risk is assessed: emergency contacts are provided, and the patient is discharged.
    - 6b: If moderate to severe risk is assessed: referral or admission to local psychiatric care is arranged if necessary.
8. The completed questionnaires are mailed to the study center.
9. A €25 voucher is sent to the clinician for distribution to the patient.
10. A receipt confirming the voucher delivery is sent back to the study center.
