# Supplementary material for: Efficient Online Recruitment of Patients With Depressive Symptoms Using Social Media: Cross-Sectional Observational Study
Source: JMIR Ment Health. 2025 Jun 3;12:e65920. doi: 10.2196/65920 (PMC12174873; doi:10.2196/65920)

Multimedia Appendix #2: Creative material used for image and video advertisement

Image	Title	Description
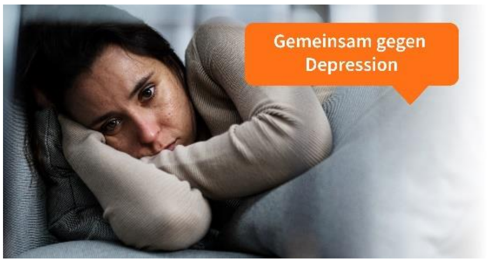	Sad Woman	Sad woman lying on the bed looking into camera – gray theme with orange textbox ("together against depression")
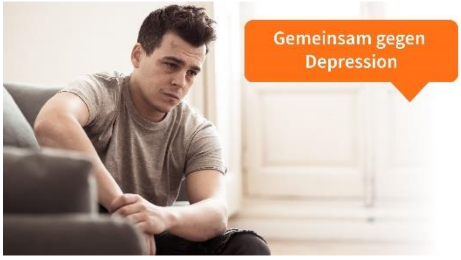	Sad Man	Sad man sitting on the couch looking downwards – sepia theme with orange textbox ("together against depression")
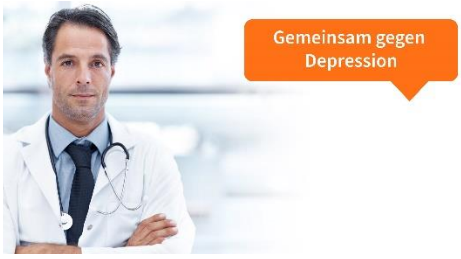	Senior Doctor	Senior male doctor looking into the camera with folded arms – white and blue theme with orange textbox ("together against depression")
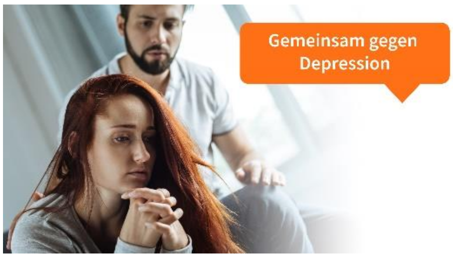	Couple 1	Couple with woman in front looking concerned and sad – grey theme with orange textbox ("together against depression")
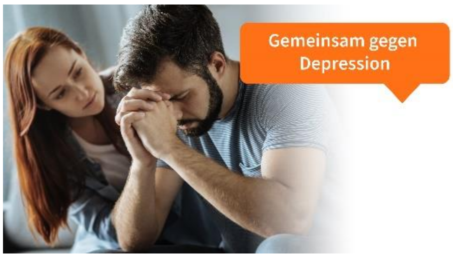	Couple 2	Couple with man in front looking concerned and sad – grey theme with orange textbox ("together against depression")

Exemplary TikTok mock-up of the video ad as medical doctor (left) and as layperson (right)

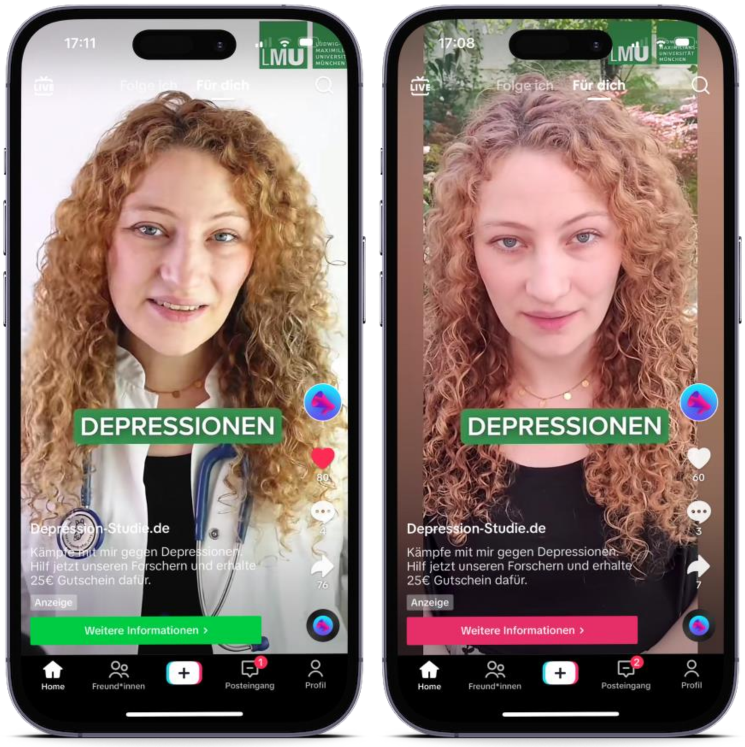

Supplement: Multimedia Appendix 2 [file mental_v12i1e65920_app2.pdf]
