# Supplementary material for: Efficient Online Recruitment of Patients With Depressive Symptoms Using Social Media: Cross-Sectional Observational Study
Source: JMIR Ment Health. 2025 Jun 3;12:e65920. doi: 10.2196/65920 (PMC12174873; doi:10.2196/65920)
Supplement: Multimedia Appendix 3 [file mental_v12i1e65920_app3.pdf]

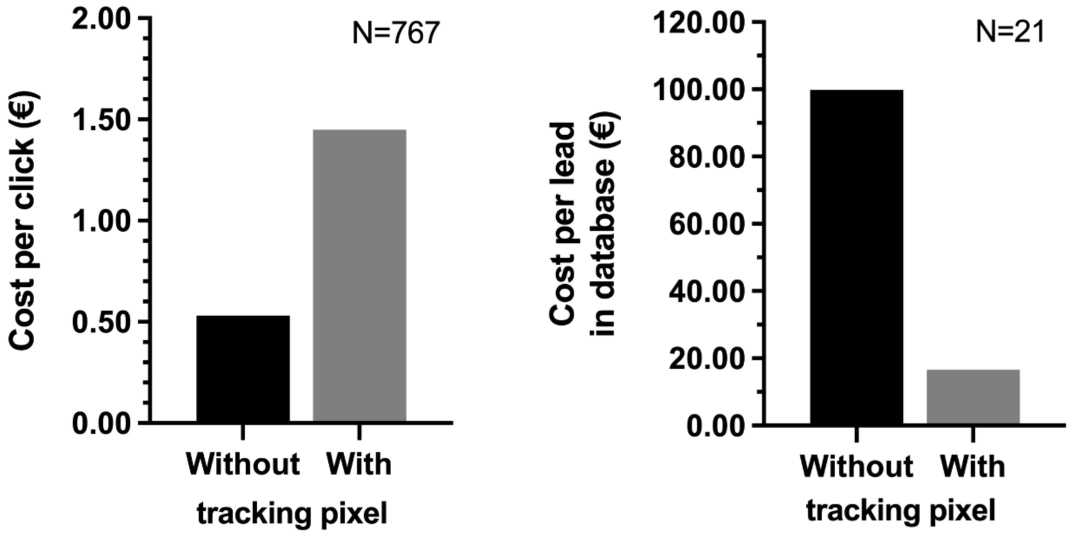


Figure 1. Results of 2 similar advertisements (€300 budget each) with and without using the Facebook tracking pixel to improve advertisement targeting and lower the costs per lead.
